# Nitrogen deposition cancels out exotic earthworm effects on plant‐feeding nematode communities

**DOI:** 10.1111/1365-2656.12660

**Published:** 2017-04-03

**Authors:** Yuanhu Shao, Weixin Zhang, Nico Eisenhauer, Tao Liu, Yanmei Xiong, Chenfei Liang, Shenglei Fu

**Affiliations:** ^1^ Key Laboratory of Geospatial Technology for the Middle and Lower Yellow River Regions (Henan University)Ministry of EducationCollege of Environment and PlanningHenan UniversityKaifeng475004China; ^2^ Key Laboratory of Vegetation Restoration and Management of Degraded EcosystemsSouth China Botanical GardenChinese Academy of SciencesGuangzhou510650China; ^3^ German Centre for Integrative Biodiversity Research (iDiv) Halle‐Jena‐LeipzigDeutscher Platz 5e04103LeipzigGermany; ^4^ Institute of BiologyLeipzig UniversityJohannisallee 2104103LeipzigGermany; ^5^ University of the Chinese Academy of SciencesBeijing100049China; ^6^ Research Institute of Tropical ForestryChinese Academy of ForestryGuangzhou510520China; ^7^ Zhejiang Provincial Key Laboratory of Carbon Cycling in Forest Ecosystems and Carbon SequestrationZhejiang A & F UniversityLin'an311300China

**Keywords:** above‐ground–below‐ground linkages, below‐ground biotic interactions, earthworm invasion, nitrogen deposition, plant‐feeding nematodes, soil nematodes

## Abstract

1. The activity and spread of exotic earthworms often are spatially correlated with N deposition because both arise from human activities. Exotic earthworms, in turn, can also greatly affect soil abiotic and biotic properties, as well as related ecological processes. Previous studies showed, for example, that earthworms can counteract the detrimental effects of plant‐feeding nematodes on plant growth. However, potential interactive effects of N deposition and exotic earthworms on ecosystems are poorly understood.
2. We explored the changes in density of plant‐feeding nematodes in response to the presence of exotic earthworms, and whether these changes are altered by elevated N deposition in a two‐factorial field mesocosm experiment at the Heshan National Field Research Station of Forest Ecosystem, in southern China.
3. Our results show that earthworm addition marginally significantly increased the density of exotic earthworms and significantly increased the mass of earthworm casts. The total density of plant‐feeding nematodes was not significantly affected by exotic earthworms or N deposition. However, exotic earthworms tended to increase the density of plant‐feeding nematode taxa that are less detrimental to plant growth (r‐strategists), while they significantly reduced the density of more harmful plant‐feeding nematodes (K‐strategists). Importantly, these earthworm effects were restricted to the ambient N deposition treatment, and elevated N deposition cancelled out the earthworm effect. Although exotic earthworms and N deposition interactively altered foliar N : P ratio in the target tree species, this did not result in significant changes in shoot and root biomass in the short term.
4. Overall, our study indicates that N deposition can cancel out exotic earthworm‐induced reductions in the density of harmful plant‐feeding nematodes. These results suggest that anthropogenic N deposition can alter biotic interactions between exotic and native soil organisms with potential implications for ecosystem functioning.

## Introduction

Understanding how soil biota responds to global change is critical for predicting the consequences for ecosystem functioning (Bardgett & Wardle 2010). Atmospheric nitrogen (N) deposition is an important component of global change (Galloway *et al*. 2004), which is likely to affect soil biota and the functions they drive (Treseder 2008). N inputs have increased dramatically due to fertilizer application and fossil fuel burning (Vitousek *et al*. 1997b), and N emissions and deposition rates are predicted to double from 2010 to 2050 (Galloway *et al*. 2004; Phoenix *et al*. 2012). The mean N wet deposition over China increased by approximately 25% from the 1990s to 2000s with the highest levels in southern China (Jia *et al*. 2014). As a consequence, ecological interactions in the soil might be changed by altered nutrient availabilities and productivity levels (Högberg *et al*. 2010; Eisenhauer *et al*. 2012a,b), making some ecosystems more vulnerable to biological invasions (Burke & Grime 1996; Fenn *et al*. 2003). Biological invasions, in turn, can substantially alter biological interactions and ecosystem processes (Vitousek 1990; Vitousek *et al*. 1997a). Most previous studies on N deposition have focused on above‐ground ecosystem responses and found, for instance, that N deposition may threaten plant diversity (Bobbink *et al*. 2010; Isbell *et al*. 2013). By contrast, although soil biodiversity is key for the functioning of terrestrial ecosystems (Bardgett & van der Putten 2014; Wall, Nielsen & Six 2015), below‐ground responses, especially changes in below‐ground biotic interactions, to N deposition are understudied (Gan, Zak & Hunter 2013, 2014). Some previous studies have shown that elevated atmospheric N deposition can alter soil microbial and faunal communities (Boxman *et al*. 1998; Xu *et al*. 2009; Zak *et al*. 2011; Eisenhauer *et al*. 2012a,b; Gan, Zak & Hunter 2013, 2014). However, how these compositional changes translate into altered below‐ground interactions and functions is unclear.

There often is a close spatial correlation of N deposition and earthworm activity linked to high anthropogenic activities (Gilliam 2006). In fact, anthopogenic degradation of the native vegetation has facilitated the expansion of exotic earthworms in southern China (Zhang *et al*. 2005), a trend which has been observed in many ecosystems world‐wide (Hendrix *et al*. 2008). The invasion of exotic earthworms, in turn, can have substantial effects on the composition and functioning of above‐ and below‐ground communities (Bohlen *et al*. 2004; Frelich *et al*. 2012; Craven *et al*. 2016). Despite the fact that elevated N deposition and exotic earthworm invasion often co‐occur and may interact with each other in influencing ecosystems, potential interaction effects on ecosystem processes remain rarely studied.

Soil nematodes are powerful indicators of changes in soil food web structure as well as soil functions, and they have manifold effects on ecosystems (Bongers 1990; Ferris, Bongers & de Goede 2001). Many plant‐feeding nematodes have net negative effects on ecosystem functions because they can decrease primary productivity by feeding on plant roots or root hairs (Ferris & Bongers 2006; Neher 2010). It is estimated that reductions in plant production caused by nematode herbivory range from 5·8 to 12·6% in grasslands (Ingham & Detling 1984) to 1·4–10% in agricultural systems (Sohlenius, Boström & Sandor 1988). Based on life‐history strategy, nematode families can be classified along a colonizer–persister (c‐p) scale. This scale ranges from one (early colonizers of new resources) to five (persisters in undisturbed habitats). Thus, the c‐p value represents life‐history characteristics associated with *r*‐ and *K*‐selection, respectively. Nematodes with a c‐p of 1 are *r*‐selected or colonizers with short generation times, large population fluctuations and high fecundity. Nematodes with a c‐p of 5 are *K*‐selected or persisters, produce few offspring and generally appear later in succession. Thus, low and high c‐p weights correspond to taxa relatively tolerant and sensitive to ecological disturbance, respectively (Bongers & Bongers 1998; Bongers & Ferris 1999). On the other hand, nematodes belonging to different c‐p‐groups may also differ in their effects on ecosystem processes. In fact, plant‐feeding nematodes belonging to the c‐p2 group mainly consist of small tylenchids and are called ‘plant‐associated nematodes’. These nematodes mostly have minor effects on plant growth and crop yield (Yeates 1999, 2007). By contrast, plant‐feeding nematodes belonging to the c‐p3‐5 group often decrease plant growth and cause crop yield loss (Yeates 1999).

Previous studies showed that earthworms can counteract the detrimental effects of plant‐feeding nematodes on plant growth (Wurst *et al*. 2008; Lohmann, Scheu & Müller 2009; Loranger‐Merciris *et al*. 2012). However, these studies did not report if the reduction of negative nematode effects on plant growth in the presence of earthworms was due to compositional shifts in the nematode community and the decrease of groups higher on the c‐p scale. Furthermore, a recent review paper summarized that earthworms usually facilitate plant growth, but the positive effects of earthworms become weaker at high N fertilizer application levels (>30 kg N ha^−1^ year^−1^) (van Groenigen *et al*. 2014). This indicates that earthworms may have significant effects on nematode communities and plant growth, but that these effects potentially depend on the N availability in the system.

Here we explored the changes in the density of plant‐feeding nematodes in response to the presence of exotic earthworms, and whether these changes are altered by elevated N deposition in a field mesocosm experiment. Plant‐feeding nematodes were classified into two groups: mostly harmless c‐p2 nematodes and harmful c‐p3‐5 nematodes). We hypothesized that N deposition in southern China (>30 kg N ha^−1^ year^−1^) could weaken the positive effects of exotic earthworms on plant growth. We explored if potential context‐dependent effects of exotic earthworms on plant growth are paralleled by earthworm‐induced changes in plant‐feeding nematodes, particularly in c‐p3‐5 nematodes. Investigating how plant‐feeding nematodes respond to the interactive effects of increasing N deposition and exotic earthworm activity allowed us to better understand below‐ground biotic interactions as affected by anthropogenic environmental alterations as well as subsequent plant growth responses.

## Materials and methods

### Site description

This study was conducted at the Heshan National Field Research Station of Forest Ecosystem (112°50′E, 22°34′N), which is located in the middle of Guangdong Province, Southern China. The climate in this region is subtropical monsoon with a hot, humid summer and a cold, dry winter. From 2004 to 2009, mean annual precipitation was 1534 mm, and mean annual temperature was 22·5 °C. The soil is an Acrisol (FAO 2006). Atmospheric N deposition in precipitation is about 43·1 ± 3·9 kg N ha^−1^ year^−1^, with a 1 : 1 ratio for NH_4_^+^ to NO_3_^−^ (Zhang *et al*. 2012). The *Acacia auriculaeformis* plantation used in this study was established in 1984. In June 2009, the mean diameter at breast height of the *A. auriculaeformis* trees was 17·2 cm. The canopy coverage was about 50%. The main understorey native plant species in this plantation are *Evodia lepta*,* Dicranopteris dichotoma*,* Rhodomyrtus tomentosa*,* Litsea cubeba* and *Ilex asprella*.

### Experimental design

Experimental sites were established under the canopy of the *A. auriculaeformis* plantation in December 2007. The experimental sites were divided into four blocks, and N application and earthworm addition treatments were assigned randomly to four equally sized plots (1 × 2 m = 2 m^2^) within each block. As a result, the experiment had a two‐factorial design (in randomized blocks; no split‐plots). The treatments were: (i) control (CK, no earthworm, no nitrogen addition), (ii) earthworm addition (E), (iii) nitrogen addition (60 kg N ha^−1^ year^−1^) (N) and (iv) earthworm addition plus nitrogen addition (NE). An 80‐cm‐deep trench was dug around each plot to prevent intrusion of roots from the outside. PVC boards (0·5 cm thick, 2 m long and 1 m wide) were then inserted into the vertical cuts to further isolate the plots; the boards extended to the bottom of the trench and 20 cm above the soil surface to prevent earthworms from moving between plots (Liu & Zou 2002; Eisenhauer *et al*. 2009). Understorey plants were removed by hand in all plots before treatments were applied. Notably, re‐growing understorey plants were removed from the experimental plots throughout the course of the experiment, because nematode abundances can be strongly controlled by the understorey plant community (De Long *et al*. 2016). Afterwards (in May 2008), we planted seven saplings of *E. lepta* per plot. In May 2009, the average diameter at the sapling base was 1·6 cm, the average height was 1·0 m and the average canopy of saplings was 0·7 m × 0·9 m. The saplings covered about 75% of the plot area.

From January 2008 to May 2009, electroshocking, a non‐destructive method, was used to reduce earthworm numbers once per month in all plots (Liu & Zou 2002; Szlavecz *et al*. 2013). All earthworms that appeared at the soil surface were picked and put out of the plots. We found two major earthworm groups, i.e., pheretimoid (including the genera *Amynthas* and *Metaphire*, both are native species) and *Pontoscolex corethrurus* (endogenic earthworm, the only exotic species) at our study site (Zhang *et al*. 2005). Pheretimoid earthworms are sensitive to electroshocking compared to *P. corethrurus* (W. Zhang & Y. Shao, pers. obs.). Thus, most pheretimoid earthworms were excluded by electroshocking. In contrast, *P. corethrurus* respond slowly to electroshocking, and thus some of the individuals may have remained in the soil. In May 2009, 2012, 2013 and 2014, we collected specimens of the *P. corethrurus*, which is widespread in tropical and subtropical regions in China (Zhang *et al*. 2005). The collected earthworms were washed in tap water and then added to the surface of the soil at the rate of 100 individuals m^−2^ at each earthworm addition event to compensate for a potential decrease in density over time and to standardize earthworm densities across treatments; this is about the average earthworm density in tropical forests (Lapied & Lavelle 2003; Marichal *et al*. 2012). As a result, the electroshock treatment reduced the population density of *P. corethrurus* in the ‘control (CK)’ plots, and earthworm addition achieved a higher population density of *P. corethrurus* in the ‘worm (E)’ plots.

Additionally, we simulated increased atmospheric nitrogen (N) deposition by performing a nitrogen addition treatment. Specifically, ammonium nitrate (NH_4_NO_3_) was weighed and dissolved in 500 ml tap water for each plot, then NH_4_NO_3_ solutions were sprayed bimonthly to the plots with a backpack sprayer starting from January 2012 to May 2014 (60 kg N ha^−1^ year^−1^; i.e. the atmospheric N input was increased by c. 150%). At each N application, each plot without N addition received 500 mL tap water.

### Soil and plant sampling and analyses

Soil samples were taken from plots before treatment application and soil pH, total soil N, soil organic carbon were measured according to Liu (1996). Four soil cores (5 cm diameter, 10 cm depth) were collected from each plot in June 2013 and June 2014. Litter was removed from soil surface before soil samples were taken. Visible roots in the soil samples were removed as quickly as possible. The soil cores from each plot were used for nematode community analysis. The soil samples taken in 2014 were also used to analyse total soil N according to Liu (1996), and soil microbial biomass N was determined based on the method described by Vance, Brookes & Jenkinson (1987). We examined the dry mass of earthworm cast on the soil surface from each plot in December 2013 and June 2014 to assess earthworm activity (Zaller & Arnone 1997). Additionally, we examined the density of exotic earthworms by sampling and hand‐sorting two 30 × 30 × 40 cm soil samples from each plot in June 2014. Meanwhile, the fine roots of *E. lepta* (diameter less than 1 mm) were picked out for fine root biomass analyses.

Nematodes were extracted from 50 g of fresh soil using Baermann funnels for each composite soil sample (Barker 1985). After fixation in 4% formalin solution, nematodes were counted under an inverted microscope, and the first 100 individuals encountered were identified to genus or family level and classified into trophic groups (plant‐feeding nematodes, bacterial‐feeding nematodes, fungal‐feeding nematodes, predators and omnivores) (Yeates *et al*. 1993) and into 16 nematode colonizer‐persistent (c‐p) and trophic group combinations (H2, H3, H4, H5, Ba1, Ba2, Ba3, Ba4, Fu2, Fu3, Fu4, P3, P4, P5, Om4 and Om5) representing five trophic groups (herbivore, bacterivore, fungivore, predator and omnivore) and c‐p values (Bongers 1990; Bongers & Bongers 1998). All nematodes were identified when the nematode number was lower than 100 individuals in a sample. The overall number of c‐p2 and c‐p3‐5 plant‐feeding nematodes was estimated according to their proportion in the 100 identified individuals. Because the aim of this study was to focus on the interactions between free‐living plant‐feeding nematodes and exotic earthworms, only genera as ascribed to the plant‐feeding trophic group were analysed in the following.

Many studies have indicated that an excessive input of N to soils can significantly deplete nutrient base cations (i.e., Ca^2+^, Mg^2+^, K^+^ and Na^+^) and causes toxic metal ions (i.e. Al^3+^, Fe^3+^ and Mn^2+^) to accumulate in soils (Gundersen, Schmidt & Raulund‐Rasmussen 2006; Bowman *et al*. 2008; Lucas *et al*. 2011), which could affect the performance of earthworms as well as the rates of other soil biological processes. Soil exchangeable cations were measured using the methods described by Hendershot, Lalande & Duquette (2007). Air‐dried soil samples were extracted in 0·1 M BaCl_2_ solutions, and concentrations of exchangeable Ca, Mg, K, Na, Al, Fe and Mn ions in extracts were determined with an inductively coupled plasma optical emission spectrometer (ICP‐OES; Perkin Elmer, Waltham, MA, USA). Soil base saturation (BS, %) was calculated as that percentage of the CEC represented by base cations (Ca, Mg, K and Na).

Foliar samples were collected from the mature canopy of *E. lepta* trees for N and phosphorus (P) analysis because foliar N : P ratios can provide information about potential nutrient limitation (Güsewell 2004; Elser *et al*. 2010). Samples were dried for 72 h at 70 °C, and dried samples were ground to a fine powder with a grinder, and homogenized by passing samples through a 150 μm screen. N concentration was measured using the Kjeldahl method (Bremner & Mulvaney 1982). P concentration was measured photometrically after samples were digested with nitric acid (HNO_3_).

### Estimation of incremental increase in plant biomass

In October 2013, we sampled naturally regenerated *E. lepta* around the plots for the parametrization of allometric equations of plant growth. When the basal diameter of *E. lepta* was less than 1 cm, we estimated plant biomass according to the basal diameter and the height of *E. lepta* because the equation based on basal diameter and height of *E. lepta* explained more variability in biomass. On the contrary, when basal diameter of *E. lepta* was greater than 1 cm, we estimated plant biomass according to the diameter at breast height and the height of *E. lepta* because the equation based on diameter at breast height and height of *E. lepta* explained more variability in biomass (Table S1, Supporting Information). The basal diameter of *E. lepta* in January 2012 and the diameter at breast height (d.b.h.) of *E. lepta* in June 2014 were measured. The estimation of plant biomass based on allometric equations for *E. lepta* is:

$$\begin{array}{cc} & W_{\mathrm{above}-\mathrm{ground}(g)}=0\;\cdot \;8246{\left(D^{2}H\right)}^{0\cdot 7595}\left(n=19,R^{2}=0\;\cdot \;99\right),\\ & W_{\mathrm{below}-\mathrm{ground}}=0\;\cdot \;1545{\left(D^{2}H\right)}^{0\cdot 7688}\left(n=20,R^{2}=0\;\cdot \;92\right) \end{array}$$

where D (cm) is the basal diameter of *E. lepta* and H (cm) is the height of *E. lepta*.

$$\begin{array}{cc} & W_{\mathrm{above}-\mathrm{ground}(\mathrm{kg})}=0\;\cdot \;0547{\left(D^{2}H\right)}^{0\cdot 8819}\left(n=25,R^{2}=0\;\cdot \;94\right),\\ & W_{\mathrm{below}-\mathrm{ground}}=0\;\cdot \;0142{\left(D^{2}H\right)}^{0\cdot 8302}\left(n=26,R^{2}=0\;\cdot \;86\right) \end{array}$$

where D (cm) is the d.b.h. of *E. lepta* and H (m) is the height of *E. lepta*.

### Statistical analysis

One‐way ANOVA was used to compare the differences of soil pH, total soil N and soil organic carbon across plots before treatment application (replicate block as error term). Two‐way repeated‐measures ANOVA (N deposition and earthworm addition as main factors, replicate block as error term) was used to compare the effects of the experimental treatments and their interaction on the dry mass of earthworm casts and the density of plant‐feeding nematodes. Two‐way ANOVA was employed to compare the effect of N deposition, earthworm addition and their interaction on the density of exotic earthworms, the increment of plant biomass, total soil N, soil microbial biomass N, soil Al^3+^ concentration, soil base saturation (BS, %), foliar N : P ratios and fine root biomass (replicate block as error term). Levene's test was performed to test for homogeneity of variance. Tukey (Tukey's honestly significant difference) tests were used to compare the effects among treatments when interactions between N deposition and earthworm addition were significant. Statistical significance was determined at *P* < 0·05. ANOVAs were performed using R version 2.15.1 (R Development Core Team 2009). To meet the assumptions of parametric statistical tests (e.g., normal distribution), the data on total plant‐feeding nematodes, c‐p2 plant‐feeding nematodes and c‐p3‐5 plant‐feeding nematodes were ln (x) transformed before performing ANOVAs.

## Results

### Soil physico‐chemical characteristics before the start of the experiment

Soil physico‐chemical characteristics (pH, total N, soil organic carbon) were similar among plots before treatment application (Table 1).

**Table 1:** Soil pH, soil organic carbon (SOC) and total soil N (TN) concentrations across plots before treatment application in a field mesocosm experiment

Treatment	pH	SOC (%)	TN (g kg^−1^)
CK	3·87 ± 0·07	2·26 ± 0·09	1·91 ± 0·16
N	3·94 ± 0·09	2·44 ± 0·13	2·04 ± 0·19
E	3·88 ± 0·02	2·37 ± 0·05	2·06 ± 0·18
NE	3·83 ± 0·03	2·21 ± 0·07	1·69 ± 0·09
Summary of ANOVA			
Treatment	*F* = 0·15, *P* = 0·93	*F* = 1·32, *P* = 0·32	*F* = 1·03, *P* = 0·42

CK, control; N, nitrogen addition; E, earthworm addition; NE, nitrogen addition plus earthworm addition. Data are means ± SE (*n* = 4).

### Earthworm treatments

**Figure 1:**
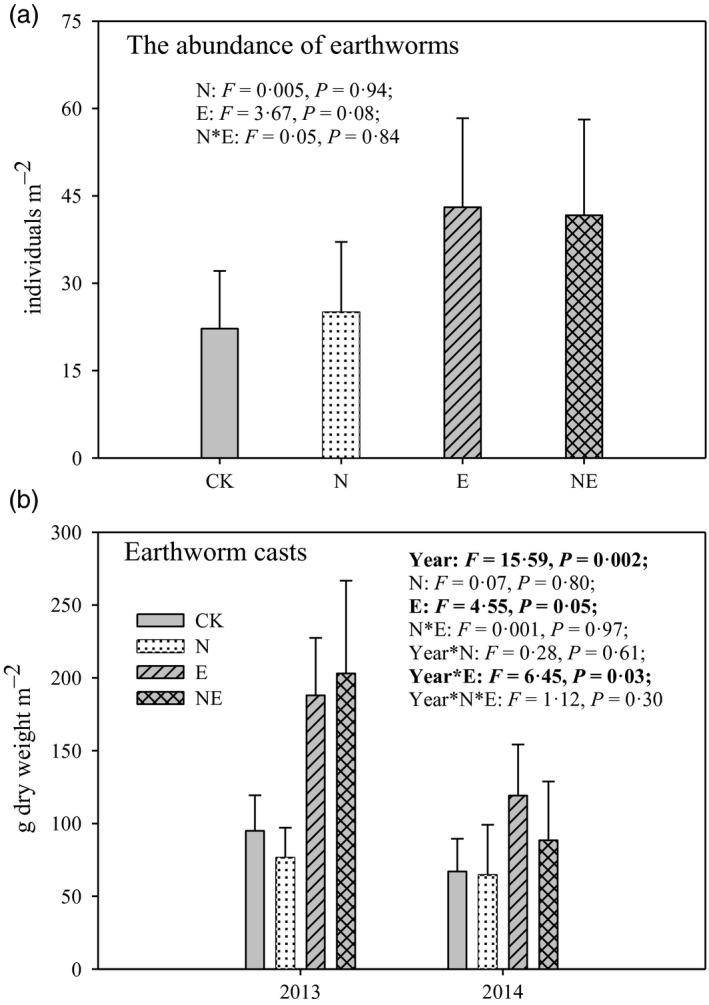
The density of exotic earthworms and the dry mass of earthworm casts in control (CK), nitrogen addition (N), earthworm addition (E) and nitrogen addition plus earthworm addition (NE) treatments in a field mesocosm experiment. Data are means + SE (*n* = 4). (a) Treatment effects from the two‐way ANOVA. (b) Treatment and year effects from two‐way repeated‐measures ANOVA. Results of Tukey's HSD *post hoc* tests (*P* = 0·05) are not provided because interaction effects in the ANOVA were not significant.

Earthworm addition marginally significantly increased the density of exotic earthworms (*P* = 0·08; Fig. 1a). Furthermore, earthworm addition significantly increased the mass of earthworm casts (*P* = 0·05; Fig. 1b) and there was an interactive effect between time and earthworm addition (Year × earthworm interaction effect: *P* = 0·03; Fig. 1b).

### Responses of plant‐feeding nematodes

**Figure 2:**
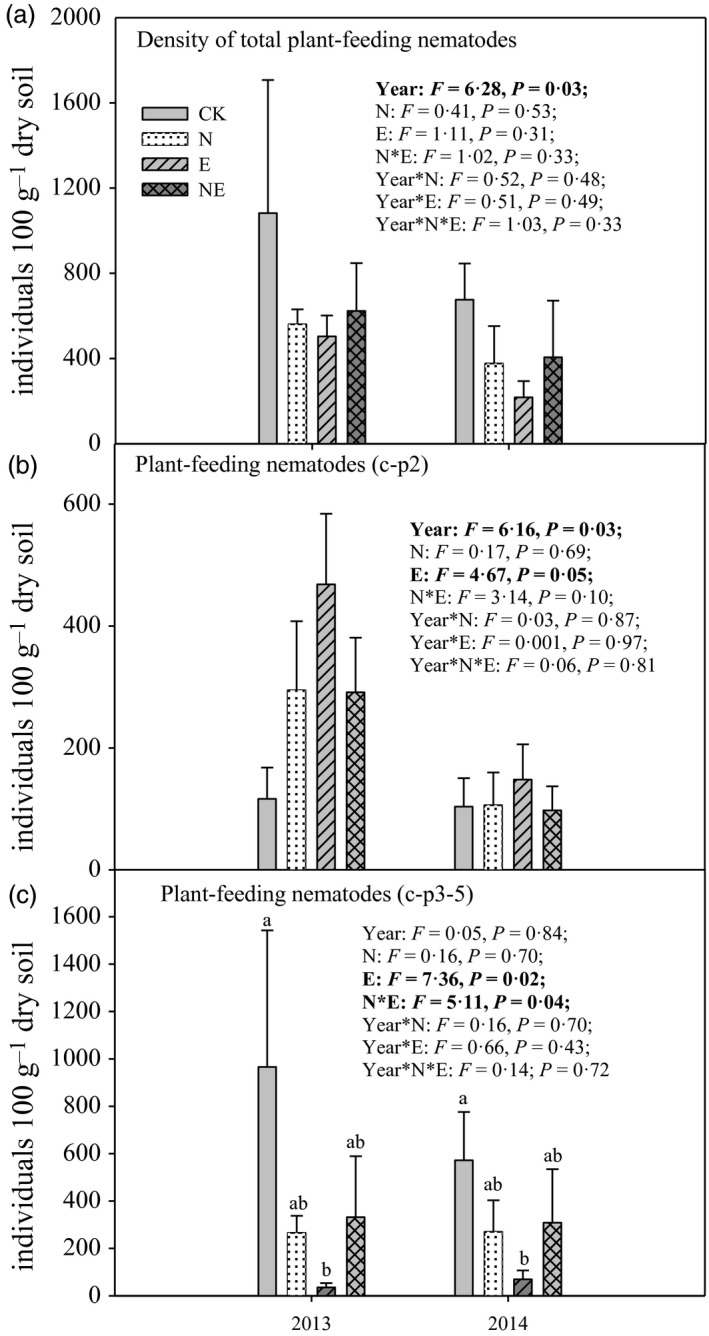
The density of total plant‐feeding nematodes, plant‐feeding c‐p2 nematodes and c‐p3‐5 nematodes in control (CK), nitrogen addition (N), earthworm addition (E) and nitrogen addition plus earthworm addition (NE) treatments in a field mesocosm experiment. Data are means + SE (*n* = 4). Treatment and year effects from two‐way repeated‐measures ANOVA are provided in each subpanel. Bars in a particular sampling time sharing the same superscript letter were not significantly different at *P* = 0·05 (Tukey). Results of Tukey's HSD *post hoc* tests (*P* = 0·05) are provided when interaction effects in the ANOVA were significant.

The density of total plant‐feeding nematodes did not change in response to N and earthworms addition (Fig. 2a). However, the presence of exotic earthworms significantly increased the density of plant‐feeding nematodes belonging to the c‐p2 group (*P* = 0·05; Fig. 2b), whereas it significantly decreased the density of plant‐feeding nematodes belonging to the c‐p3‐5 group (*P* = 0·02; Fig. 2c). Additionally, although no significant main effects of N deposition on soil nematodes were found, N deposition altered the exotic earthworm effect on c‐p3‐5 nematodes (N deposition × earthworm interaction effect: *P* = 0·04): the negative effect of exotic earthworms on c‐p3‐5 nematodes disappeared in the context of N deposition (Fig. 2c).

### Incremental increase in plant biomass, fine root biomass and foliar N : P ratios

**Figure 3:**
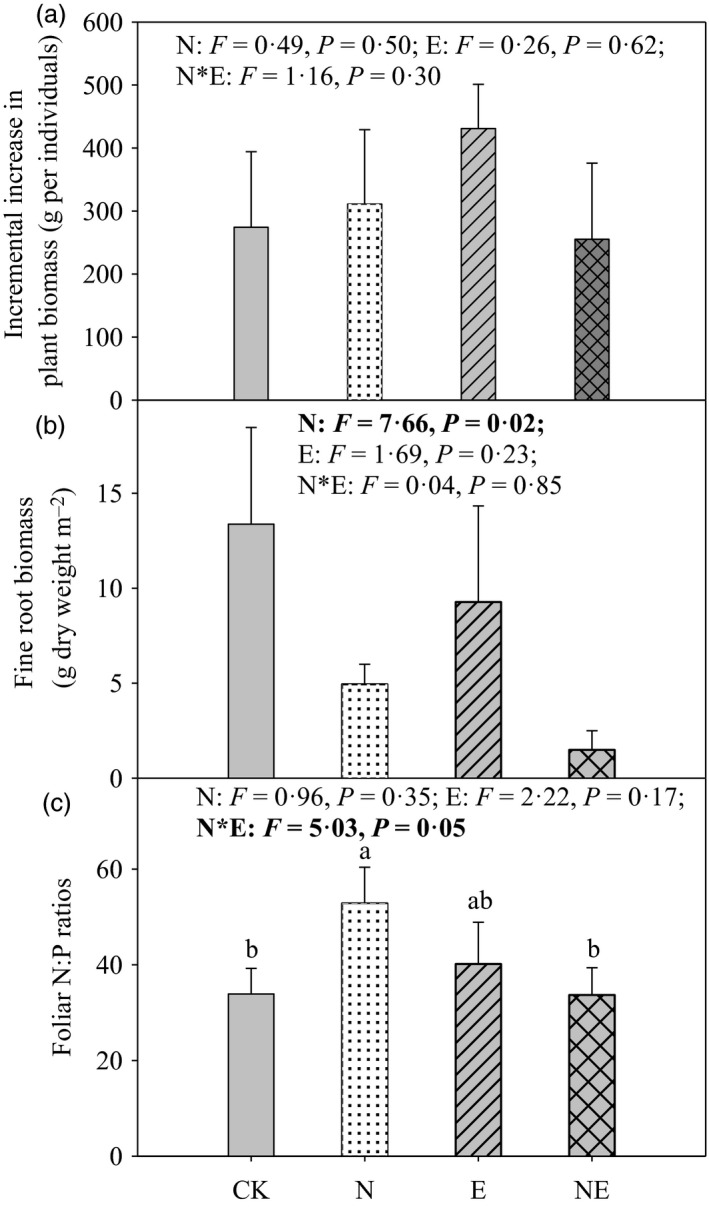
Incremental increase in plant biomass, plant fine root biomass and foliar N : P ratios in control (CK), nitrogen addition (N), earthworm addition (E) and nitrogen addition plus earthworm addition (NE) treatments in a field mesocosm experiment. Data are means + SE (*n* = 4). Treatment effects from the two‐way ANOVA are provided in each subpanel. Bars sharing the same superscript letter were not different significantly at *P* = 0·05 (Tukey). Results of Tukey's HSD *post hoc* tests (*P* = 0·05) are provided when interaction effects in the ANOVA were significant.

Although no significant differences of increments in plant biomass occurred among treatments (there was only a non‐significant trend of higher increment in plant biomass in the presence of exotic earthworms without N addition; Fig. 3a), N deposition decreased the biomass of fine roots (*P* = 0·02, Fig. 3b). Moreover, foliar N : P ratios were relatively high (>30) in the studied system and depended on a significant interaction effect of N deposition and earthworm presence (Fig. 3c, *P* = 0·05). While N deposition alone tended to increase foliar N : P ratios (Fig. 3c, *P* = 0·10), this effect was weakened in the presence of exotic earthworms (Fig. 3c, *P* = 0·08).

### Soil Al^3+^ concentrations and soil base saturation

The soil Al^3+^ concentration and soil base saturation were similar among treatments (Fig. S1). However, Al^3+^ concentrations tended to increase (*P* = 0·09; Fig. S1a), and soil base saturation tended to decrease in response to N deposition (*P* = 0·08; Fig. S1b).

## Discussion

Understanding the linkages between above‐ and below‐ground compartments is one of the most important issues in terrestrial ecology (Kardol & Wardle 2010). Very little is known about below‐ground biotic interactions as well as their dependencies on different global change agents (Eisenhauer *et al*. 2012a,b), but these interactions are critical for driving plant‐soil feedback effects and related ecosystem processes (Kardol *et al*. 2015). In contrast to previous studies (e.g., Wurst *et al*. 2008; Lohmann, Scheu & Müller 2009; Loranger‐Merciris *et al*. 2012), exotic earthworms did not significantly affect the total density of plant‐feeding nematodes in our study. However, exotic earthworms reduced the density of c‐p3‐5 plant‐feeding nematodes, which are most detrimental for plant growth, while they increased the density of c‐p2 plant‐feeding nematodes. Most importantly, earthworm effects on plant‐feeding nematodes were found only at ambient levels of N deposition. Generally, plant‐feeding nematodes can affect plant growth and related functions through feeding on plant roots or root hairs (Ferris & Bongers 2006; Neher 2010). Plant‐feeding nematodes belonging to the cp3‐5 group mainly feed on deeper cell layers in the roots than nematodes lower on the c‐p scale do (Bongers & Bongers 1998) because they can use their stronger stylet to penetrate into the cortex more deeply and form larger cavities, which potentially are more susceptible to lead to the infection of soil‐borne pathogens and cause stronger root damage (Back, Haydock & Jenkinson 2002). The density of cp3‐5 plant‐feeding nematodes may decrease in the presence of earthworms due to enhanced tolerance of plants to antagonists (Blouin *et al*. 2005; Lafont *et al*. 2007) or physical disturbance by the burrowing activity of earthworms (Eisenhauer 2010). By contrast, c‐p2 plant‐feeding nematodes (mostly smaller tylenchids in the present study, see Table S2) are less plant host‐specific, or perhaps more dependent on rhizosphere secretions and soil micro‐organisms (Yeates 1999, 2007). Therefore, c‐p2 plant‐feeding nematodes may have benefitted from the slight but non‐significant positive effects of earthworms on plant growth in the present study. These results show that the effects of earthworms on plant‐feeding nematodes depend on the life‐history traits of the nematodes (here represented by the c‐p scale) and soil nutrient levels.

Earthworms, as ecological engineers in soil ecosystems (Blouin *et al*. 2013), play important roles in increasing plant biomass production, e.g. through enhancing nutrient availability and changing biotic interactions in the soil (Scheu 2003; van Groenigen *et al*. 2014). The biocontrol of pests and diseases could be a possible pathway by which earthworms can positively affect plant growth (Brown, Edwards & Brussaard 2004). For example, earthworms can enhance plant health as well as growth and decrease the need for cysteine protease production by improving availability of oxygen and/or water to the roots (Brown *et al*. 1999). In addition, earthworms release hormone‐like chemicals from casts, which can enhance plant health and improve the resistance of plant to the plant‐parasitic nematodes (Blouin *et al*. 2005; Lafont *et al*. 2007).

In addition to earthworm effects on soil biota, earthworms promote soil nitrogen mineralization and thus facilitate plant growth (Scheu 2003; Dechaine *et al*. 2005; Baker 2007; van Groenigen *et al*. 2014). A recent meta‐analysis suggested that the effects of earthworms on plant growth in studies with low fertilizer N application rates (<30 kg N ha^−1^ yr^−1^) were stronger than in studies with high fertilizer N application rates (>30 kg N ha^−1^ yr^−1^) (van Groenigen *et al*. 2014). Thus, elevated atmospheric N deposition could change the effects of exotic earthworms on plant‐feeding nematodes and have subsequent consequences for plant growth.

The simulated N deposition rate in our study reached 60 kg N ha^−1^ yr^−1^; although this currently represents a relatively high level of nitrogen deposition, it is expected to be realized in this region in the near future. Thus, due to the relatively high level of nitrogen deposition, we expected to detect a reduction of the strength of the earthworm effect on plant growth, because the positive effects of earthworms on soil nitrogen mineralization could be weakened or overtrumped (van Groenigen *et al*. 2014). However, earthworm effects on plant growth were non‐significant both under ambient and elevated N conditions in the present study. In contrast to our expectation, we did not find any effects of N deposition on plant‐feeding nematodes or plant growth, which indicates that N availability was not the limiting factor in the studied system. Indeed, plant phosphorus (P) limitation may play a major role when foliar N : P ratios are >20 (Güsewell 2004). In the present study, foliar N : P ratios were relatively high (>30), indicating a strong phosphorus limitation in the studied system. Furthermore, N inputs are likely to alter soil nutrient conditions, such as soil acidification, losses of base cations and Al mobilization (Matson *et al*. 1999; Chen *et al*. 2015). However, we did not find any significant effects of N addition on the total soil N and soil microbial biomass N in the present study (Table S3); it is possible that plant‐available N is taken up and utilized by the plant community quickly in the studied region or N addition affected the ecological processes related to nitrogen but had no effects on total soil N or soil microbial biomass N. High Al^3+^ concentrations in soils can cause oxidative stress and cell damage in earthworms (Li *et al*. 2014), with potential negative effects on earthworm performance. The trends of marginally increased soil Al^3+^ concentration and decreased soil base saturation at elevated N deposition indicate that net neutral effects of enhanced N availability on plant growth may have been partly due to lower soil P availability in response to higher soil Al^3+^ concentrations (Matson *et al*. 1999). Therefore, elevated N deposition may have exacerbated phosphorus limitation and increased soil Al^3+^ concentration in our experiment, and this could have weakened the effects of exotic earthworms on nutrient mineralization and plant growth. Our results indicate that anthropogenic N deposition can alter earthworm‐induced changes in plant‐feeding nematode communities, which has the potential to influence above‐ground–below‐ground interactions.

Anthropogenic activities can alter the relative importance and interplay among different regulating processes of plant growth (Dighton 2014). Thus, elevated N deposition is often considered as environmental pollution (Ochoa‐Hueso & Manrique 2011), and may have detrimental effects on plant growth, e.g. when decreasing biodiversity (Isbell *et al*. 2013). Here, the role of altered biotic interactions in the soil needs more scientific attention. Biological invasions pose a major threat to many ecosystems around the globe (Murphy & Romanuk 2014). Particularly, the invasion of exotic earthworms has attracted increasing scientific attention due to their significant effects on native biodiversity (Craven *et al*. 2016), ecosystem processes (Bohlen *et al*. 2004) and soil communities (Eisenhauer 2010). The present study shows, however, that effects of invasive earthworms on soil communities and plant nutrition depend on environmental conditions, such as N deposition rates. These findings are in line with previous studies showing that exotic earthworm performance (Eisenhauer *et al*. 2014) and effects on plant communities (Eisenhauer *et al*. 2012a,b) depend on other environmental change drivers, complicating predictions of exotic earthworm impacts on native ecosystems. Thus, our findings suggest that anthropogenic N deposition can alter biotic interactions in soils and has the potential to affect plant health and growth, with significant consequences for above–below‐ground interactions. Therefore, the consideration of interactions among soil organisms may improve the mechanistic understanding of above‐ and below‐ground interactions as well as plant growth responses in the context of multiple environmental change factors, such as elevated N deposition and earthworm invasion.

## Authors' contributions

S.F., W.Z., Y.S. and Y.X. were involved in study design, data interpretation and paper preparation; Y.S., T.L. and C.L. collected the data; Y.S. and N.E. analysed the data; Y.S. and N.E. led the writing of the manuscript. All authors contributed critically to the drafts and gave final approval for publication.

## Data accessibility

Data available from the Dryad Digital Repository https://doi.org/10.5061/dryad.9tq18 (Shao *et al*. 2017).

## Supporting information


**Table S1.** Allometric equations of plant growth for *Evodia lepta* at Heshan National Field Research Station of Forest Ecosystem in October 2013.
**Table S2.** Density of plant‐feeding nematodes in control (CK), nitrogen addition (N), earthworm addition (E) and nitrogen addition plus earthworm addition (NE) treatments in a field mesocosm experiment.
**Table S3.** Total soil N (TN) and soil microbial biomass N in control (CK), nitrogen addition (N), earthworm addition (E) and nitrogen addition plus earthworm addition (NE) treatments in a field mesocosm experiment.
**Fig. S1.** Soil Al^3+^ and base saturation (BS) in control (CK), nitrogen addition (N), earthworm addition (E) and nitrogen addition plus earthworm addition (NE) treatments in a field mesocosm experiment.
