# Supplementary material for: Nitrogen deposition cancels out exotic earthworm effects on plant‐feeding nematode communities
Source: J Anim Ecol. 2017 Apr 3;86(4):708–17. doi: 10.1111/1365-2656.12660 (PMC5484995; doi:10.1111/1365-2656.12660)
Supplement: Supplementary file 1 — Table S1. Allometric equations of plant growth for Evodia lepta at Heshan National Field Research Station of Forest Ecosystem in October 2013. Table S2. Density of plant‐feeding nematodes in control (CK), nitrogen addition (N), earthworm addition (E) and nitrogen addition plus earthworm addition (NE) treatments in a field mesocosm experiment. Table S3. Total soil N (TN) and soil microbial biomass N in control (CK), nitrogen addition (N), earthworm addition (E) and nitrogen addition plus earthworm addition (NE) treatments in a field mesocosm experiment. Fig. S1. Soil Al3+ and base saturation (BS) in control (CK), nitrogen addition (N), earthworm addition (E) and nitrogen addition plus earthworm addition (NE) treatments in a field mesocosm experiment. [file JANE-86-708-s001.docx]

**Supporting Information**

Table S1 Allometric equations of plant growth for *E. lepta* at Heshan National Field Research Station of Forest Ecosystem in October 2013.

| Model | n values | R^2^ | Basal diameter |
| --- | --- | --- | --- |
| W_aboveground_ = 0.8246(D^2^H)^0.7595^ | 19 | 0.9946 | < 1 cm |
| W_belowground_ = 0.1545(D^2^H)^0.7688^ | 20 | 0.9169 | < 1 cm |
| W_aboveground_ = 0.0547(D^2^H)^0.8819^ | 25 | 0.9357 | > 1 cm |
| W_belowground_ = 0.0142(D^2^H)^0.8302^ | 26 | 0.856 | > 1 cm |

Note: When the basal diameter of *E. lepta* is less than 1 cm, D (cm) is the basal diameter of *E. lepta* and H (cm) is the height of *E. lepta*. When the basal diameter of *E. lepta* is greater than 1 cm, D (cm) is the diameter at breast height (d.b.h.) of *E. lepta* and H (m) is the height of *E. lepta*.

Table S2 Density of plant-feeding nematodes in control (CK), nitrogen addition (N), earthworm addition (E), and nitrogen addition plus earthworm addition (NE) treatments in a field mesocosm experiment. Data are means ± SE (n=4).

| Treatment | Functional  Group (family/genus) | Density (individuals 100g^-1^ dry soil) | |
| --- | --- | --- | --- |
|  |  | 2013 | 2014 |
| CK | c-p2 (*Boleodorus, Tylenchidae*) | 116.4±51.4 | 103.7±46.8 |
|  | c-p3-5 (*Pratylenchus*, *Trichodorus*, *Longidorus*, *Xiphinema*) | 966.2±576.5 | 572.2±203.7 |
| N | c-p2 (*Boleodorus, Aglenchus*) | 294.7±113.2 | 106.1±53.5 |
|  | c-p3-5 (*Pratylenchus*, *Trophurus*, *Paratrichodorus*, *Trichodorus*, *Longidorus*) | 265.5±72.0 | 270.1±132.6 |
| E | c-p2 (*Boleodorus, Basiria, Tylenchidae*) | 468.5±115.7 | 148.1±57.7 |
|  | c-p3-5 (*Pratylenchus*, *Helicotylenchus,* *Trichodorus*) | 35.3±18.4 | 69.3±37.5 |
| NE | c-p2 (*Boleodorus*) | 291.3±89.6 | 97.6±39.5 |
|  | c-p3-5 (*Pratylenchus*, *Criconema, Trophurus, Paratrichodorus*, *Longidorus*, *Xiphinema*) | 331.6±257.5 | 308.3±226.3 |

Table S3 Total soil N (TN) and soil microbial biomass N in control (CK), nitrogen addition (N), earthworm addition (E), and nitrogen addition plus earthworm addition (NE) treatments in a field mesocosm experiment. Data are means ± SE (n=4).

| Treatment | TN (g kg^-1^) | Soil microbial N (mg kg^-1^) |
| --- | --- | --- |
| CK | 1.66 ± 0.06 | 70.45 ± 8.39 |
| N | 1.75 ± 0.08 | 86.72 ± 19.95 |
| E | 1.76 ± 0.16 | 57.07 ± 12.34 |
| NE | 1.53 ± 0.14 | 70.42 ± 12.66 |
| Summary of ANOVA |  |  |
| N | *F* = 0.35, *P* = 0.57 | *F* = 1.12, *P* = 0.31 |
| E | *F* = 0.26, *P* = 0.62 | *F* = 1.13, *P* = 0.31 |
| N * E | *F* = 1.80, *P* = 0.20 | *F* = 0.01, *P* = 0.92 |

Figure S1. Soil Al^3+^ and base saturation (BS) in control (CK), nitrogen addition (N), earthworm addition (E), and nitrogen addition plus earthworm addition (NE) treatments in a field mesocosm experiment. Data are means + SE (n=4). Treatment effects from the two-way ANOVA were provided. Results of Tukey’s HSD post hoc tests (*P* = 0.05) are not provided because interaction effects in the ANOVA were not significant.
